# Function-selective domain architecture plasticity potentials in eukaryotic genome evolution

**DOI:** 10.1016/j.biochi.2015.05.003

**Published:** 2015-12

**Authors:** Viktorija Linkeviciute, Owen J.L. Rackham, Julian Gough, Matt E. Oates, Hai Fang

**Affiliations:** a Computational Genomics Group, Department of Computer Science, University of Bristol, The Merchant Venturers Building, Bristol BS8 1UB, UK; b School of Biological Sciences, University of Edinburgh, Darwin Building, The King's Buildings, Edinburgh EH9 3BF, UK; c Centre for Computational Biology, Duke-NUS Graduate Medical School, Singapore 169857, Singapore; d Wellcome Trust Centre for Human Genetics, University of Oxford, Roosevelt Drive, Oxford OX3 7BN, UK

**Keywords:** Domain architectures, Eukaryotic genomes, Evolution, Function, FDR, false discovery rate, GO, Gene Ontology, HMM, hidden Markov models, PP, plasticity potential, SCOP, Structural Classification of Proteins, sTOL, tree of (sequenced) life

## Abstract

To help evaluate how protein function impacts on genome evolution, we introduce a new concept of ‘architecture plasticity potential’ – the capacity to form distinct domain architectures – both for an individual domain, or more generally for a set of domains grouped by shared function. We devise a scoring metric to measure the plasticity potential for these domain sets, and evaluate how function has changed over time for different species. Applying this metric to a phylogenetic tree of eukaryotic genomes, we find that the involvement of each function is not random but highly selective. For certain lineages there is strong bias for evolution to involve domains related to certain functions. In general eukaryotic genomes, particularly animals, expand complex functional activities such as signalling and regulation, but at the cost of reducing metabolic processes. We also observe differential evolution of transcriptional regulation and a unique evolutionary role of channel regulators; crucially this is only observable in terms of the architecture plasticity potential. Our findings provide a new layer of information to understand the significance of function in eukaryotic genome evolution. A web search tool, available at http://supfam.org/Pevo, offers a wide spectrum of options for exploring functional importance in eukaryotic genome evolution.

## Highlights

• A new concept to measure domain architecture plasticity potential in a genome.
• We reveal the function-selective role in eukaryotic genome evolution.
• Eukaryotic genomes expand signalling and regulations but reduce metabolism.
• We observe differential evolution between trans- and cis-acting regulations.
• We observe a unique role of channel regulators in separating eukaryotic kingdoms.

## Graphical abstract

**Figure:**
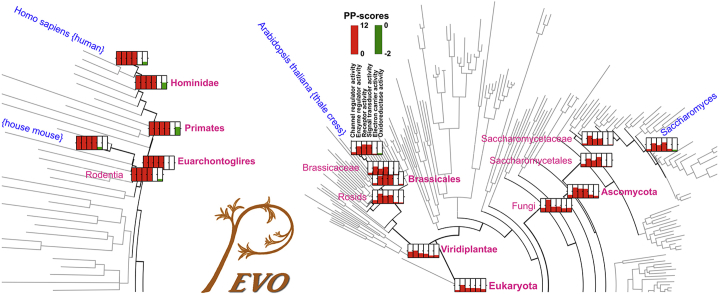

## 1 Introduction

### 1.1 The importance of protein-domain architectures in understanding genome evolution

Elucidating the importance of function in directing eukaryotic evolution is vital to explain the phenotypic diversity of observed living forms. We present a first attempt towards a systematic description and comparison of protein function over the evolution of eukaryotic proteomes. A proteome is an entire protein repertoire (encoded by a genome), and is composed of proteins comprised of structural units or domains [1]. For simplicity, hereinafter the words ‘genome’ and ‘proteome’ are used interchangeably (e.g. protein domain assignments for a genome actually means assignments for the proteome encoded by the genome). Also, the referred to in this work are those with well-defined 3D structure, although other types of domains and their functional importance have been described elsewhere [2], [3], [4], [5], [6]. As building blocks, domains are either found alone or combined to create multi-domain proteins. It is generally accepted that domains often act as functional units [7], [8] creating a basis for the complete functional repertoire for proteins. This modularity of proteins is likely favoured by evolution because it allows for combining pre-existing domains to acquire new functions [9]. The sequential order of the domains that make up a protein is referred to as its domain architecture (or ‘architecture’ in brief). Our previous analysis has shown that most extant architectures evolve from ancient architectures, and convergent/polyphyletic evolution of architectures resulting in the same architectures in eukaryotic species of different lineages is rare [10]. Furthermore, studies by others show that the evolutionary changes to architectures are more common by domain insertions than deletions, and the insertion of domains is preferred at the terminus over internally [11]. Proteins with the same or similar architectures tend to be homologous and functionally similar [12], [13]. The emergence of new architectures is thought to be a major mechanism of new functionality [14], [15]. Our recent study on the evolution of human cells suggests that the exaptation (opposed to adaptation) of existing architectures is probably a major source of cell types [16]. So far, studying domain architectures at the genome scale (both in extant and ancestral genomes) is the most realistic approach to comprehensively understand the evolutionary forces shaping eukaryotic genomes.

### 1.2 Genomic content of protein domain architectures in eukaryotic genomes

**Fig. 1:**
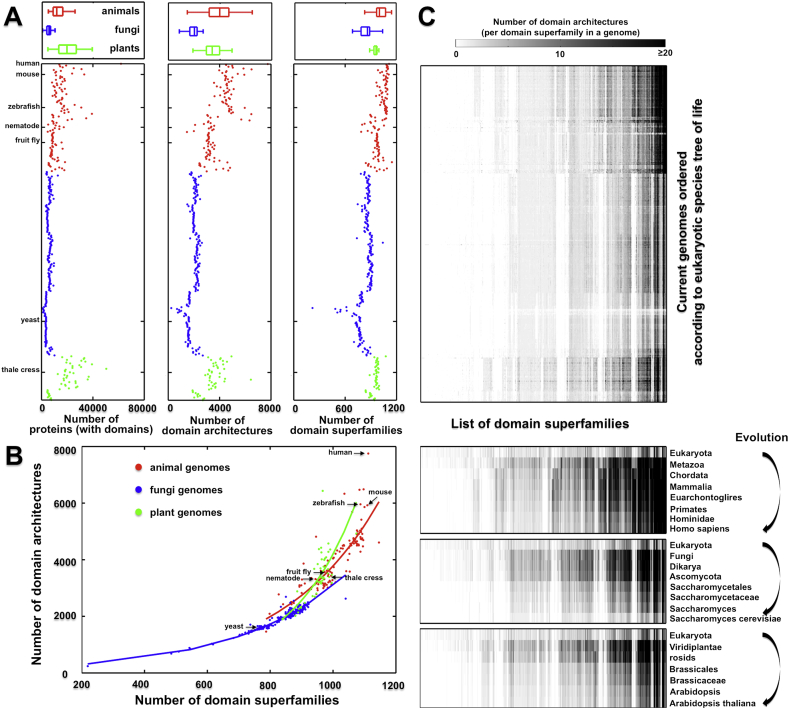
Domain superfamilies and architectures in eukaryotic genomes. (A) The total number of proteins with domain assignments (left), domain architectures (middle) and domain superfamilies (right) for eukaryotic genomes. The genomes are ordered according to the eukaryotic species tree of life, and are colour-coded in red for the animal kingdom, in blue for the fungi kingdom and in green for the plant kingdom. Also indicated are some commonly studied organisms. The box plots above show the kingdom-specific distributions. (B) The scatter plots of eukaryotic genomes with the total number of domain architectures against that of domain superfamilies. The solid curves are fitted for each of the three kingdoms. The trend emphasizes the importance of architecture diversity over superfamily diversity. (C) Heatmap depicting the number of domain architectures attached to each domain superfamily (column) and in a genome (row). The top panel is for all current/extant eukaryotic genomes, and the bottom panel is for the lineages from the eukaryotic common ancestor leading towards: Homo sapiens (human), Saccharmyces cerevisiae (yeast) and Arabidopsis thaliana (thale cress). Each row in this map forms the architecture plasticity landscape for a genome, while each column represents (in terms of an individual superfamily) similarities, differences and evolutionary changes.

The Structural Classification of Proteins (SCOP) database is a gold standard for classifying protein domains of known structure [17]. According to SCOP, a domain superfamily is defined to group together domains for which there is structural, sequence and functional evidence for a common ancestor. Hereinafter, ‘domain superfamilies’ and ‘superfamilies’ are used interchangeably. Using this definition of domains, the SUPERFAMILY database builds hidden Markov models (HMMs) for assigning domain compositions for genome sequences [18]. It provides the most comprehensive assignment of SCOP domain architectures to publicly available genome sequences [19], including those in eukaryotic genomes and their ancestral architectures reconstructed from the eukaryotic species tree of life [20]. Fig. 1 illustrates the status quo for eukaryotic genome information in the SUPERFAMILY database. Across genomes there is a remarkably similar number of superfamilies but a much higher variation for the number of architectures. On average, the number of proteins (with domains) is higher in plant genomes than in animal genomes, but the reverse is true for architectures (Fig. 1A). When plotting the architecture number against superfamily number for each of eukaryotic genomes (Fig. 1B), it becomes clear that it is the repertories of domain architectures that more closely correlate with organism complexity than protein domains. As superfamilies increase in number, architectures undergo an exponential increase, indicating that the emergence of architectures (rather than superfamilies) contributes to the organism complexity. Still, there exists the ‘G-value paradox’ (the gene/protein number is not expectedly related to the complexity [21]), even in terms of architecture number.

### 1.3 Concept of protein domain architecture plasticity potential

To better describe the relationship between genomes, superfamilies and architectures, we introduce a new concept of *‘architecture plasticity potential’*, the capacity of a domain superfamily to occur in different architectural contexts (i.e. the number of different architectures) within a genome. From this concept, architecture plasticity potential differs from one superfamily to another. The upper panel of Fig. 1C illustrates architecture plasticity potential for superfamilies across extant eukaryotic genomes. For an extant genome, most superfamilies occur only in a small number of architectures, but with a few superfamilies present in many architectures. This power-law-like pattern is similar to the previous report for domain combinations [22] and for domain architectures [23], suggesting that architecture plasticity potential is likely an intrinsic property of superfamilies (i.e. superfamily-specific). This superfamily-specific potential also differs between genomes. For a given superfamily, in general animal genomes have a higher degree of architecture diversity than plant and fungi genomes, and this potential is evolvable in a highly lineage-specific manner (the lower panel of Fig. 1C). Notably, our concept of ‘architecture plasticity’ looks similar to but is different from the previous concepts such as ‘domain versatility’ [24] and ‘domain promiscuity’ [25], [26]. The architecture plasticity is closely related to the (unique) architectural design of the proteins, while the domain versatility/promiscuity is much related to the combinatory nature of domains.

### 1.4 Opportunity for studying functional significance in eukaryotic genome evolution

Based on preliminary data present in Fig. 1 and the concept of architecture plasticity potential introduced in Section 1.3, we intend to examine dynamic changes of architecture diversity during eukaryotic evolution, not only for an individual superfamily, but also for a collection of superfamilies sharing a certain biological property (especially function). A somewhat overlooked area of research is the need for functional annotations of protein domains (even though their importance as functional units has been widely recognised). Recently, we have released the dcGO database [8], together with open-source software ‘dcGOR’ [27], providing a systematic annotation of domains using a panel of ontologies including Gene Ontology (GO) and expanding our sparse manual functional annotations [28]. This resource has been assessed in the CAFA competition [29], [30], and has been effectively utilised for cross-knowledge and cross-species studies [31]. As well as defining architecture plasticity potentials for individual superfamilies, we also generalize the definition to describe a collection of functionally related superfamilies (e.g. annotated by a GO term in the dcGO database). As such we are able to address the question of how functional information carried by protein domains influences the architectural diversity over the course of eukaryotic genome evolution.

## 2 Materials and methods

### 2.1 Genomic domain assignments and architectures

Domain assignments for sequenced genomes were obtained from the SUPERFAMILY database [32], a routinely updated resource that was initially developed for structural genomics analysis [18] but now has been extended to phylogenomics analysis [20]. At the time of writing (September 2014) SUPERFAMILY contains 437 eukaryotic proteomes and 1674 superfamilies (defined by SCOP [17] at the superfamily/evolutionary level with an evidence for a common ancestor). Each proteome is annotated using HMMs based on these superfamilies and subsequently each protein sequence is converted into a sequence of SCOP superfamily domains or gaps, i.e. the protein's domain architecture. Here we are interested in, given a genome, the potential of a superfamily to be present in different architectures. Thus we prepared a matrix of 1674 superfamilies × 437 genomes, wherein each element corresponds to the number of different architectures associated with a superfamily (in a row) that is present in a genome (in a column).

### 2.2 Ancestral genomic architectures in eukaryotic evolution

Recently, we have published the sTOL [20], a tree of (sequenced) life that provides an evolutionary context for genome-wide studies. The sTOL is a fully resolved binary tree, with each internal node either being mapped onto a known ancestral species or left unlabelled as a hypothetical unknown ancestor. Since the convergent evolution of domain architectures is rare, particularly in eukaryotes [10], we have applied Dollo parsimony [33] to reconstruct ancestral states of domain architectures for ancestral genomes in the eukaryotic part of the sTOL [20], which has also been used for estimating gene evolutionary age [34]. Similar to the extant genomic architectures in Section 2.1, we represented ancestral genomic architectures in the form of matrix, which consists of 1674 superfamilies × 436 ancestral genomes (i.e., 436 internal nodes for eukaryotic part of sTOL). The meaning of this matrix is to describe the potential of a superfamily to form different architectures in an ancestral genome.

### 2.3 Domain-centric annotations with a functional aspect

GO annotations for domain superfamilies were obtained from the dcGO database [8] which was created via statistical analysis of domain content and ontology annotations at the protein/gene level [32]. Based on domain-centric GO annotations, dcGO also contains a slim version (subset) of ontology terms. Ontology terms in the slim set are classified into four levels of increasing specificity: highly general, general, specific and highly specific. We considered domains annotated by a specific term/function as its ‘domain set’, and it is this domain set that is then used to further investigate that term/function. For instance, the set of all domains annotated by a GO term ‘enzyme regulator activity’ is considered the ‘enzyme regulator activity’ domain set, and as such is used for studying that function.

### 2.4 Quantifying plasticity potential of individual domains and domain sets in terms of architectures

The tendency for an individual domain to occur in distinct/different architectures (i.e. its architecture diversity) can be described by a ‘plasticity potential’ (PP). High PP for a domain means it is frequently found in diverse architectures, while low PP indicates a domain occurring in few distinct architectures. The number of different architectures is counted over every protein sequence present in a genome (i.e. the proteome) without using any filtering criteria. With this definition, the PP is genome-specific since the same domain may have different PP across different genomes. In this way, the PP can be used for studying the dynamic changes of architecture diversity (attached to a given domain), e.g. during eukaryotic genome evolution. In a similar way the PP for a domain set can also be estimated by calculating an average number of architectures per domain in the set. To ensure the estimate is insensitive to extreme values, we used the median (*MED*) as an averaging metric to quantify the plasticity potential *PP*_*ds*_(*g*) of a domain set (*ds*) for a given genome (*g*):

(1) $$PP_{ds}\left(g\right)=\underset{d\in ds}{MED(}N_{d})\text{,}$$

where *d*∈*ds* denotes a list of individual domains belonging to the domain set *ds* and *N*_*d*_ for the number of architectures associated with the domain *d*. To allow for comparison across genomes and across terms (i.e. domain sets), we next consider the null distribution of the *PP*_*ds*_(*g*). A null distribution was estimated using a randomization procedure, which simultaneously respects the size of the domain set, the domain repertoires present in the genome, and the collection of annotatable domains (e.g. only those annotated by one GO term or more). Specifically, we generated a random instance of domain set *ds*^*b*^, which contained the same number of domains as in *ds* but being randomly sampled from the annotatable domain repertoires present in the genome *g*. In a similar way to *PP*_*ds*_(*g*), we calculated the randomized PP for this instance, denoted as $PP_{ds}^{b}\left(g\right)$. By repeating this randomization procedure *B* times (2000 or higher), we obtained a sampling of the null distribution: $PP_{ds}^{b}\left(g\right)$, *b* = *1* … *B*. This null distribution was then used to estimate the sample mean *μ*_*ds*_(*g*), sample standard deviation *σ*_*ds*_(*g*), and the plasticity potential score (PP-score). They are formulated as:

(2) $$\mu_{ds}\left(g\right)=\frac{1}{B}\sum_{b\in B}PP_{ds}^{b}\left(g\right)\text{,}$$

(3) $$\sigma_{ds}\left(g\right)=\sqrt{\frac{1}{B-1}\sum_{b\in B}{\left[PP_{ds}^{b}\left(g\right)-\mu_{ds}\left(g\right)\right]}^{2}}\text{,}$$

(4) $$PP-score=\frac{PP_{ds}\left(g\right)-\mu_{ds}\left(g\right)}{\sigma_{ds}\left(g\right)}\text{,}$$

A PP-score of zero suggests the domains from a given domain set have the same potential to form different architectures as those domains being chosen randomly. A positive PP-score indicates the tendency of the domain set to form more different architectures than would be expected by chance, while a negative PP-score telling the tendency to form less diversified architectures as compared to random. To estimate statistical significance for the PP-score, we also calculated a P-value from the null distribution sampled above:

(5) $$P-value=\{\begin{array}{cc} \frac{1}{B}\sum_{b\in B}I\left\{PP_{ds}^{b}\left(g\right)\geq PP_{ds}\left(g\right)\right\}, & PP-score>0\\ \frac{1}{B}\sum_{b\in B}I\left\{PP_{ds}^{b}\left(g\right)\leq PP_{ds}\left(g\right)\right\}, & PP-score<0 \end{array}$$

where I{·} is an indicator function that returns 1 when meeting the inner condition, and 0 otherwise. The P-values were then corrected using the Benjamini-Hochberg derived step-up procedure for false discovery rate (FDR) to account for multiple hypothesis tests.

## 3 Results

### 3.1 Illustration of architecture plasticity potentials for a collection of related domain superfamilies

**Fig. 2:**
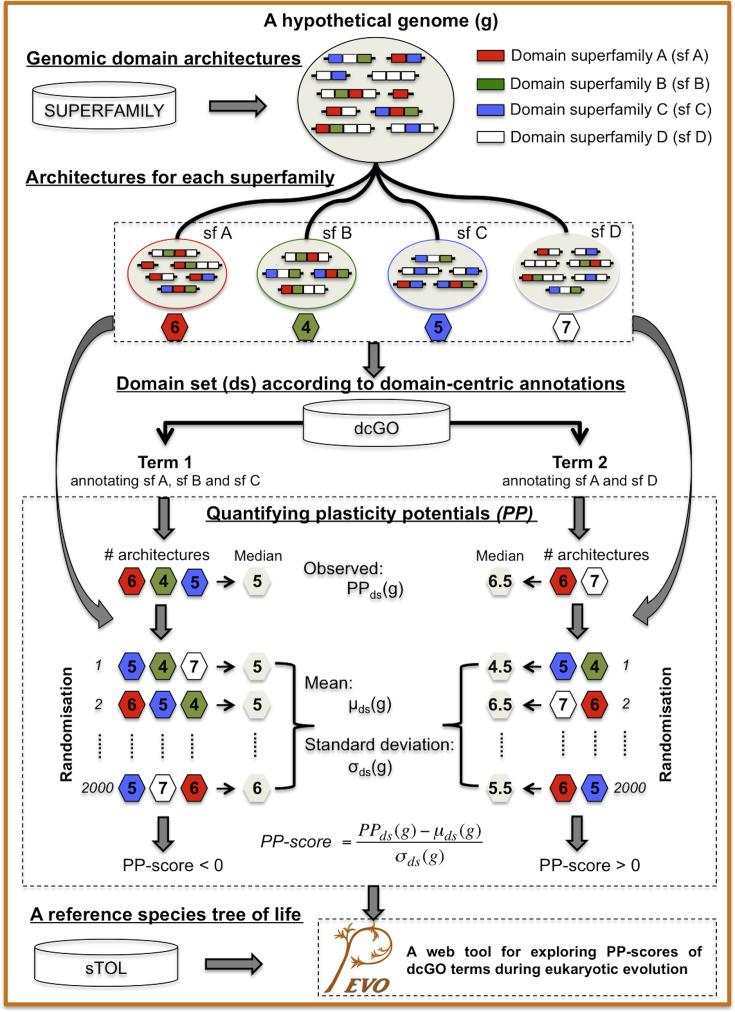
A schematic flowchart for introducing and implementing the concept of architecture plasticity potentials for a collection of related domains in a hypothetical genome.

We use a hypothetical genome (illustrated in Fig. 2) to explain the concept of plasticity potentials (PP) and the metric used to digitise such potentials. This genome (the top larger circle in Fig. 2) contains 10 distinct domain architectures that are formed by 4 different domain superfamilies *A, B, C, D* (colour-coded boxes). Following this, there are four smaller circles below, each denoting the architecture repertories/landscape of the associated superfamily. The number of architectures (colour-coded hexagons) defines the PP for an individual domain. This definition can be naturally extended to a collection of related domains annotated by an ontology term (i.e. a domain set sharing certain common characteristics). Without loss of generality, hereinafter we view an ontology term as equivalent to a domain set. As exemplified in Fig. 2, ‘Term 1’ (the left panel) is equivalent to three superfamilies *A*, *B* and *C* it annotates, and ‘Term 2’ (the right panel) to the two superfamilies *A* and *D*. The PP for a domain set is quantified by taking the median number of architectures per domain (that is, 5 for ‘Term 1’ and 6.5 for ‘Term 2’). To allow for the PP to be comparable across genomes and across terms, we further devise a new scoring metric called ‘PP-score’. The PP-score takes into account the mean and standard deviation of the PP that would be expected when randomly sampling any domain set of the same size within this genome. Using this randomisation, the negative PP-score for the ‘Term 1’ is indicative of the tendency to form less diversified architectures than by chance. Conversely, the positive PP-score for the ‘Term 2’ suggests the tendency to form more different architectures than by chance. A key point of this strategy is to use term-specific architecture plasticity potentials for correlating functions (carried by GO terms) with genome evolution. Our recent progress in building relevant databases makes this strategy feasible. The SUPERFAMILY database [32] provides protein-domain architecture annotation for all publically available sequenced proteomes and a reconstruction and annotation of the ancestral proteomes across eukaryotic evolution. The dcGO database [8] offers a wide spectrum of ontology terms describing functions and many others. The sTOL resource [20] provides the evolutionary context for exploring the dynamic changes of the PP-score during the genome evolution.

### 3.2 Systematic characterisation of molecular functional activities modulated in eukaryotic evolution

**Fig. 3:**
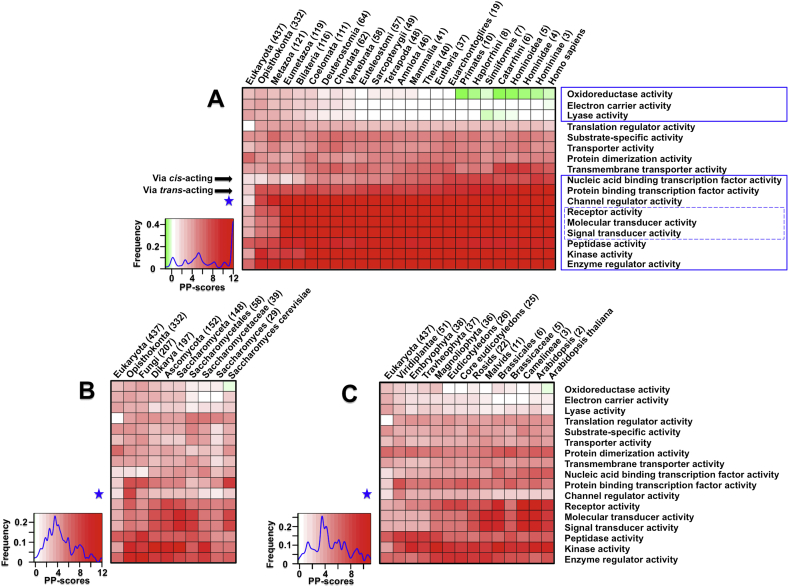
Architecture plasticity potentials of GO terms compared across three kingdoms of eukaryotic evolution. GO terms selected are conferred with different molecular activities, thus representing a wide spectrum of functions. The heatmap illustrates the plasticity potential patterns from the eukaryotic common ancestor (left-most): along the metazoan/animal lineage leading towards human (A), along the fungal lineage leading towards yeast (B), and along the Viridiplantae/plant lineage leading towards Arabidopsis (C). The colour bars on the bottom-left corner display the magnitude of PP-scores (x-axis), but also show the frequency distribution of PP-scores in each heatmap (blue curve in y-axis). The number in parenthesis indicates how many species within the clade.

The extent to which function-selective pressure operates upon genome evolution remains an open question. Here, we applied the concept introduced above to systematically address this question. To do so, we selected GO terms that are representative of different kinds of molecular functions/activities, and for each we calculated the PP-scores in all eukaryotic genomes and their ancestral genomes. Fig. 3 illustrates the patterns revealed in three kingdoms of eukaryotic life: starting from eukaryotic common ancestor (the left most) through the respective lineage leading towards human (the metazoan/animal lineage; Fig. 3A), yeast (the fungal lineage; Fig. 3B) and Arabidopsis (the plant lineage; Fig. 3C). Frequency distributions (see colour bars) show that there are overwhelmingly positive PP-scores, regardless of whether the genomes are extant or ancestral, and regardless of different kingdoms. This observation clearly suggests that functionally related domains tend to form more diverse architectures than unrelated ones (e.g. randomly chosen domains); such a tendency is most prominent along the lineage of animals compared to other kingdoms. According to the patterns observed in this lineage, we grouped GO terms into three major categories: (i) three specific terms (i.e., ‘oxidoreductase activity’, ‘electron carrier activity’ and ‘lyase activity’) which have a decreasing pattern and are exclusively associated with metabolic processes; (ii) terms describing transporter–relevant activities which show almost a flat pattern, although with some fluctuations; (iii) the rest of the terms have an overall increasing pattern, particularly at the early evolutionary history, and they are primarily associated with complex functional activities. Together, we observe domain architecture plasticity potentials in a highly function-selective manner, and the key results are further explained in details below.

#### 3.2.1 Evolution of complex functions in animals

**Fig. S1:**
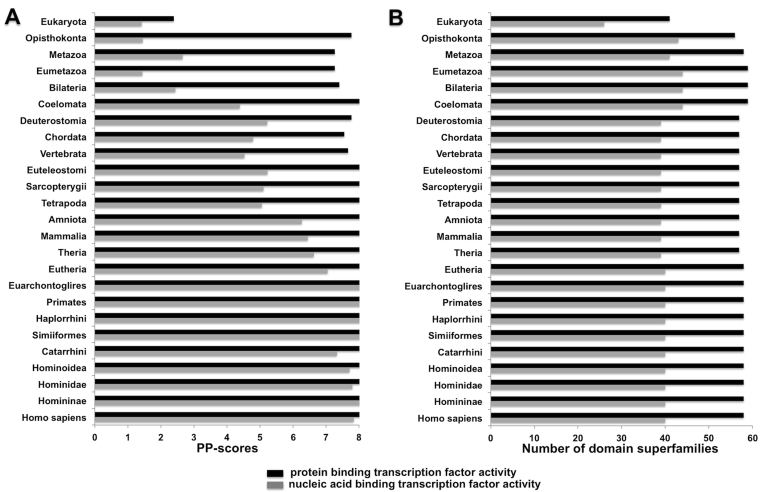
Patterns of transcription factor activities in terms of PP-scores (A) and the number of domain superfamilies (B) in the course of evolution leading towards human.

**Fig. 4:**
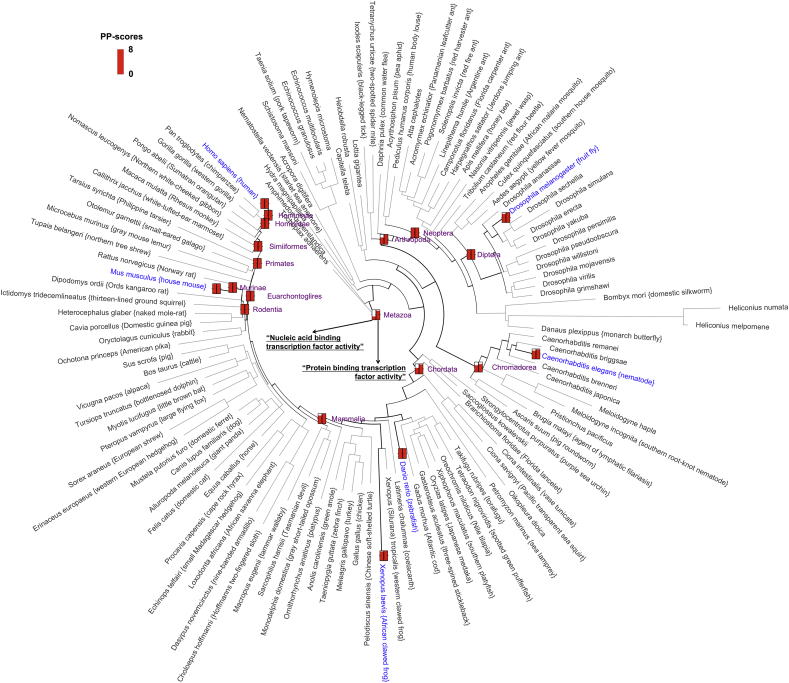
Differential patterns of transcription factor activities in terms of architecture plasticity potentials in animal evolution. Transcription factor activities have two modes for transcriptional regulation, one *cis*-acting via ‘nucleic acid binding’ and the other *trans*-acting via ‘protein binding’. Their PP-scores are visualised using side-by-side thermometers at each major branching point of the animal species tree of life (circular phylogram). For easy visualisation, only the major lineages leading to the commonly used model organisms are displayed.

Among functional processes that are increasing, three signalling-related activities (framed in dotted blue lines of Fig. 3A) experience two periods of expansion: the first at the formation of the animal-fungi common ancestor (*Opisthokonta*), and the second occurring during the shift from the animal ancestor (*Metazoa*) to its subkingdom (*Eumetazoa*). In contrast, we only observed a dramatic increase when shifting to *Opisthokonta* for activities related to channel/enzyme regulation, i.e. expansion of kinase and peptidase families. In animal evolution, the rise of multicellularity (especially the occurrence of *Bilateria*) is a key transition, for which signalling systems are a prerequisite [35]. Our data suggest that, in addition to the rise of an animal-fungi common ancestor, the diversification of signalling pathways occurred earlier than the *Bilateria*. This observation clarifies the previous view that signalling systems were likely to have arisen before the split with bilaterians (and even the metazoans) [36], and also strengthens their importance and association with the emergence of multicellularity. For transcription factor activities, there are two modes for the transcriptional regulation: *cis*-acting (binding to nucleic acids) and *trans*-acting (binding to proteins). We found that the ‘*trans*-acting’ mode has a dramatic increase in architectural plasticity potentials at the rise of the animal-fungi common ancestor; thereafter, such potentials are maintained. In sharp contrast, the ‘*cis*-acting’ mode experiences a steady increase along with increasing organism complexity. This observation is not just limited to human-specific evolution (Fig. 3A), but is universal to the evolution for other animal model organisms, such as mouse, frog, zebrafish, worm and fly illustrated in Fig. 4. Our data provides additional knowledge on transcription factor evolution in animals [37], as it implies differential evolution of transcriptional regulation. Recent high-throughput experimental data has revealed a high degree of interspecies changes in transcription factor DNA binding [38]. Consistent with this, much more varied ‘nucleic acid binding transcription factor activities’ were perhaps needed to adapt to DNA sequence changes during animal evolution. However, there seems no continued evolutionary pressure for ‘protein binding transcription factor activities’ once established in earlier history. Notably, such differential patterns (Fig. S1A) cannot be explained by the creation/deletion of domain superfamilies, as changes in the number of domain superfamilies are similar for both transcription factor activities (Fig. S1B). Therefore, it appears to be unlikely that the evolutionary pressure operates directly on the ‘domain quantity’ but acts in the ‘architectural context’ of the functions instead.

#### 3.2.2 A unique role of channel regulators in separating three kingdoms of eukaryotic life

**Fig. 5:**
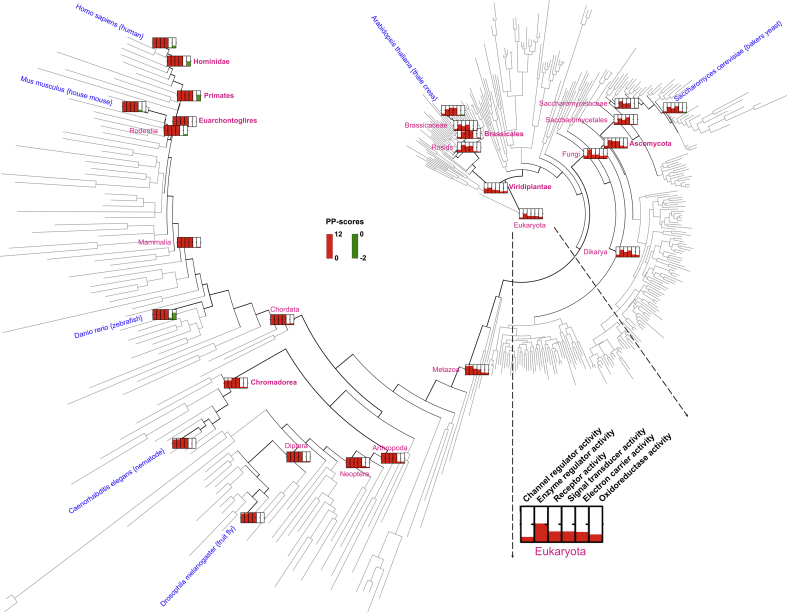
A unique pattern for channel regulator activity in terms of architecture plasticity potentials in eukaryotic evolution. The circular phylogram displays the eukaryotic species tree that is labelled with the commonly used model organisms and their major common ancestors. The bottom-right corner illustrates the zoomed-in thermometers measuring the PP-scores for the indicated terms in eukaryota. In addition to the term ‘channel regulator activity’, five more terms are also shown for comparison. They are ‘enzyme regulator activity’, two signalling terms ‘Receptor activity’ and ‘Signal transducer activity’, and two metabolic terms ‘electron carrier activity’ and ‘oxidoreductase activity’.

The patterns revealed in Fig. 3A in general hold true for both the fungal lineage leading towards yeast and plant lineage towards Arabidopsis (Fig. 3B and C): a decreasing pattern for metabolic activities, no changes for transport activities, an increasing pattern for complex functional activities (particularly signalling). However, we did observe differences, largely in the amplitude of the patterns. The strongest difference is ‘channel regulator activity’. Once diversified from the animal-fungi common ancestor, the pattern was sustained along the animal lineage leading towards human but not along the fungal lineage towards yeast. A similar result was also observed for the plant lineage; it was only required for the first appearance of the plant kingdom but not thereafter. Unlike the metabolic and complex activities, this pattern is unique to channel regulator activity only (Fig. 5). It is logical to speculate that channel regulators (e.g. calcium, chloride, potassium, sodium and other ions) mainly function to drive the separation of three kingdoms of eukaryotic life, continue to push animal evolution, but appear to be less diversified for the continuation of the other two kingdoms.

### 3.3 Web search tool enabling hypothesis-driven research

All findings described above are drawn from a functional perspective, but in principle the same analysis can be applied to any subject, such as the evolution of diseases and phenotypes. To facilitate attempts in these promising directions, we provide a web search tool ‘Pevo’ (http://supfam.org/Pevo). It offers a wide spectrum of ontology terms, representing over 5000 research topics not just on functions, but also on phenotypes and diseases, in the hope of exploring their evolutionary importance over the eukaryotic tree of life. The users can pick up any terms of their interest, and similarly in Fig. 3, Fig. 4, the side-by-side term comparisons are graphically visualised within the circular phylogram. By default, the whole eukaryotic phylogenetic tree is used, highlighting only those commonly used model organisms (and their major common ancestors). The users can add other species or switch to the kingdom-specific view. We anticipate that the concepts of architecture plasticity potentials, together with this tool being freely open to the research community, will provide a road map to systematically test (and generate) hypotheses with respect to the relationships between evolution and function or indeed any other ontology terms.

## 4 Discussion and conclusions

We introduce and implement the concept of architecture plasticity potentials to better understand the role of function in eukaryotic genome evolution. We observe that the way nature shapes architecture diversity is not random. It is more economical from an evolutionary viewpoint to re-use existing protein domains by diversifying the repertoire of architectures (in a function-selective manner), than it is to diversify by creating new domains and deleting existing ones. Probably under the pressure of increasing organism complexity, the diversity of architectures harbouring superfamilies that are involved in complex functions increases at the cost of the diversity of architectures containing metabolism-related superfamilies. This is particularly prominent in animal evolution, suggesting that there is a robust signal underlying our observations (i.e. of complex functions *vs*. metabolism). Because of this robustness, we are also able to uncover the evolutionary importance of signalling systems for multicellularity, differential evolution between *trans*-acting and *cis*-acting transcriptional regulation, and the uniqueness of channel regulators in the formation of the three kingdoms. Although some of our findings are as expected, great care needs to be taken when making interpretations. Take these two terms as examples: one is the metabolism-related term ‘electron carrier activity’, and the other the signalling-related term ‘molecular transducer activity’. Both terms have almost the same positive PP-scores in the eukaryotic common ancestor. Along the evolutionary lineage to human, there is a steady decrease in the PP-score (even becoming negative) for the metabolism term, whereas the opposite trend is observed for the signalling term. This strong function-selective pattern cannot be simply explained by saying that this metabolism is no longer needed by human. Instead, as a basic activity it is still essential but just gives way to the need for increased signalling, seen as a reduction in the architectural repertoire. It is a matter of balance: in human we see much more architecture diversity being formed for signalling in place of metabolism; but in the eukaryotic common ancestor we see a relative equilibrium. The plasticity potential is a layer of information revealing the functional selection, which can be imagined as ‘invisible software’, operating on the architectural design of the proteins, which can be imagined as the ‘visible hardware’.

## Conflict of interest

The authors declare that no competing interests exist.
